# Hexa­kis­(μ_3_-1-methyl­thio­urea-κ^3^ *S*:*S*:*S*)hexa­kis­[iodidocopper(I)]

**DOI:** 10.1107/S1600536812043437

**Published:** 2012-10-24

**Authors:** Saeed Ahmad, Muhammad Mufakkar, Islam Ullah Khan, Hoong-Kun Fun, Abdul Waheed

**Affiliations:** a Department of Chemistry, University of Engineering and Technology, Lahore 54890, Pakistan; b Department of Chemistry, Government College of Science, Wahdat Road, Lahore, Pakistan; c Department of Chemistry, Government College University, Lahore, Pakistan; d X-ray Crystallography Unit, School of Physics, Universiti Sains Malaysia, 11800 USM, Penang, Malaysia

## Abstract

The title compound, [Cu_6_I_6_(C_2_H_6_N_2_S)_6_], was obtained from the reaction of copper(I) iodide with *N*-methyl­thio­urea (Metu) in equimolar amounts in acetonitile. The complex consists of two six-membered trinuclear Cu_3_S_3_I_3_ cores that combine through triply bridging Metu, forming a hexa­nuclear core which has -3 symmetry. The Cu^II^ atom is coordinated by three S atoms of Metu and one iodide ion in a distorted tetra­hedral geometry. The crystal structure is stabilized by N—H⋯I hydrogen bonds and cuprophilic inter­actions [Cu⋯Cu = 3.0264 (9) Å].

## Related literature

For crystal structures of copper(I) complexes of thio­urea-type ligands, see: Ahmad *et al.* (2010 ▶); Bowmaker *et al.* (2009 ▶); Li *et al.* (2005 ▶); Lobana *et al.* (2003 ▶, 2005 ▶); Khan *et al.* (2007 ▶); Mufakkar *et al.* (2007 ▶, 2009 ▶, 2011 ▶); Stocker *et al.* (1997 ▶); Zoufala *et al.* (2007 ▶). For van der Waals radii and cuprophilic inter­actions, see: Siemeling *et al.* (1997 ▶); Singh *et al.* (1997 ▶).
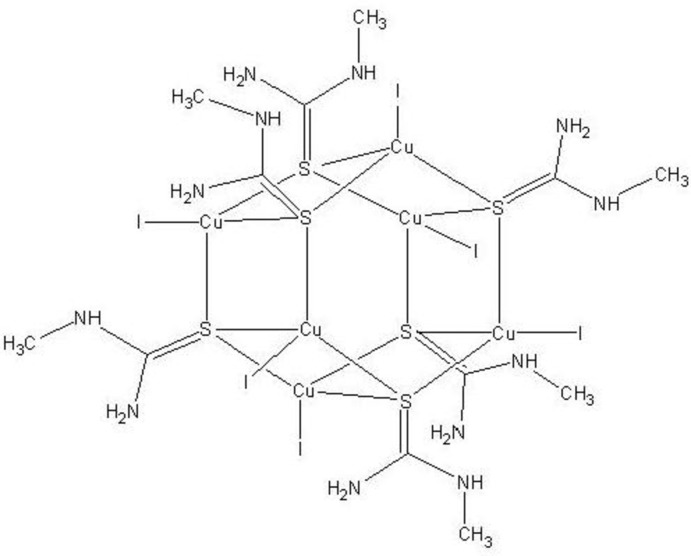


## Experimental

### 

#### Crystal data

[Cu_6_I_6_(C_2_H_6_N_2_S)_6_]
*M* *_r_* = 1683.65
Trigonal, 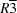

*a* = 21.7517 (1) Å
*c* = 7.6269 (1) Å
*V* = 3125.11 (5) Å^3^
*Z* = 3
Mo *K*α radiation
μ = 7.79 mm^−1^
*T* = 296 K
0.28 × 0.15 × 0.14 mm

#### Data collection

Bruker SMART APEXII CCD area-detector diffractometer
Absorption correction: multi-scan (*SADABS*; Bruker, 2008 ▶) *T*_min_ = 0.179, *T*_max_ = 0.338
14898 measured reflections
1995 independent reflections
1649 reflections with *I* > 2σ(*I*)
*R*_int_ = 0.035

#### Refinement

*R*[*F* ^2^ > 2σ(*F* ^2^)] = 0.027
*wR*(*F* ^2^) = 0.061
*S* = 1.06
1995 reflections
65 parameters
H-atom parameters constrained
Δρ_max_ = 1.32 e Å^−3^
Δρ_min_ = −1.64 e Å^−3^

### 

Data collection: *APEX2* (Bruker, 2008 ▶); cell refinement: *SAINT* (Bruker, 2008 ▶); data reduction: *SAINT*; program(s) used to solve structure: *SHELXTL* (Sheldrick, 2008 ▶); program(s) used to refine structure: *SHELXTL*; molecular graphics: *SHELXTL*; software used to prepare material for publication: *SHELXTL* and *PLATON* (Spek, 2009 ▶).

## Supplementary Material

Crystal structure: contains datablock(s) I, global. DOI: 10.1107/S1600536812043437/rz5016sup1.cif

Structure factors: contains datablock(s) I. DOI: 10.1107/S1600536812043437/rz5016Isup2.hkl

Additional supplementary materials:  crystallographic information; 3D view; checkCIF report

## Figures and Tables

**Table 1:** Hydrogen-bond geometry (Å, °)

*D*—H⋯*A*	*D*—H	H⋯*A*	*D*⋯*A*	*D*—H⋯*A*
N1—H1*N*1⋯I1^i^	0.83	2.95	3.744 (3)	161
N1—H2*N*1⋯I1	0.80	2.90	3.698 (4)	173
N2—H1*N*2⋯I1^ii^	0.80	2.95	3.756 (3)	177

Symmetry codes: (i) 
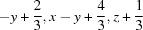
; (ii) 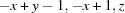.

**Fig. 1.**
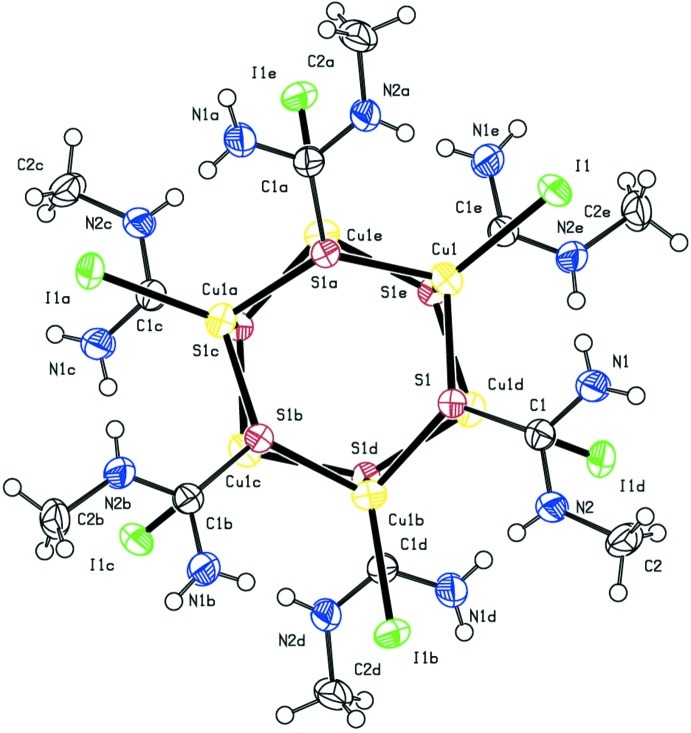
Molecular structure of the title compound with displacement ellipsoids drawn at 50% probability level. Symmetry codes: (a) 1-y, 2+x-y, z; (b) -1-x+y, 1-x, z; (c) -x, 2-y, -z; (d) -1+y, -x+y, -z; (e) 1+x-y, 1+x, -z.

## supplementary crystallographic information

### Comment

Copper(I) complexes with thiones possess a variety of structures ranging from mononuclear three- or four- coordinate species with trigonal planar and tetrahedral Cu(I) respectively to hexameric species with pseudo-four-coordinated geometry (Ahmad *et al.* 2010; Bowmaker *et al.*, 2009; Li *et al.*, 2005; Lobana *et al.*, 2003, 2005; Khan *et al.* 2007; Mufakkar *et al.*, 2007, 2009, 2011; Stocker *et al.*, 1997; Zoufala *et al.*, 2007). In some cases mononuclear units further aggregate to form polymeric structures, for example, [Cu_6_(PyT)_6_I_6_]n (where Pyt = pyridine-2-thione) (Li *et al.*, 2005; Lobana *et al.*, 2003, 2005). The present report describes the structure of a hexameric copper(I) complex, iodido(*N*-methylthiourea)copper(I), that is characterized by significant copper-copper interactions.

The structure of the title complex is shown in Figure 1. The complex is hexanuclear consisting of six [Metu-Cu—I] units, associated through sulfur atoms of *N*-methylthiourea. Three copper(I) iodides and three Metu ligands are combined through bridging sulfur atoms to form a six-membered trinuclear core, Cu_3_S_3_I_3_. Two six-membered trinuclear cores combine *via* µ_3_-sulfur atoms of Metu to form the centrosymmetric hexanuclear core, Cu_6_S_6_I_6_. Each copper within the complex is coordinated to three sulfur atoms of *N*-methylthiourea and with one iodide as a terminal ligand adopting a distorted tetrahedral geometry. The angles around Cu vary over the range 98.22 (5)–122.56 (3) °. The Cu—S bond distances are unequal; two are short (2.3164 (10) and 2.3210 (10) Å) and one is long (2.6057 (13) Å). However, they are within the range (2.30–2.60 Å) of the Cu—S bond distances found in other complexes. All of the Cu—I distances are equal (2.5379 (5) Å) and are in agreement with the values reported in the literature. The hexanuclear structure is supported by significant intermolecular N—H···I hydrogen bonding (Table 1) and Cu···Cu interactions. The Cu···Cu distance of 3.0264 (9) Å is close to similar distances observed in other complexes. However, this value is slightly larger than the sum of the van der Waals radii of two copper atoms (2.80 Å) (Siemeling *et al.* 1997; Singh *et al.*, 1997). Similar hexanuclear core structures have been reported for [Cu_6_(Imt)_6_I_6_]n and [Cu_6_(Pyt)_6_I_6_]n (Imt = imidazolidine-2-thione and Pyt = pyridine-2-thione; Lobana *et al.*, 2003, 2005).

### Experimental

The title compund was prepared by mixing solutions of copper(I) iodide (1.0 mmol) in 10 ml acetonitrile and *N*-methylthiourea (1.0 mmol) in acetonitrile (15 ml). The mixture was stirred for half an hour and then filtered. The resulting colourless solution when allowed to stand for 24 h yielded white crystals suitable for X-ray structure analysis.

### Refinement

All H atoms were placed in calculated positions with C—H = 0.96 Å, N—H = 0.80-0.83 Å, and with *U*_iso_(H) = 1.2 *U*_eq_(N) or 1.5 *U*_eq_(C)

### Crystal data

**Table:**

[Cu_6_I_6_(C_2_H_6_N_2_S)_6_]	*D*_x_ = 2.684 Mg m^−^^3^
*M**_r_* = 1683.65	Mo *K*α radiation, λ = 0.71073 Å
Trigonal, *R*3	Cell parameters from 4617 reflections
Hall symbol: -R 3	θ = 2.2–26.6°
*a* = 21.7517 (1) Å	µ = 7.79 mm^−^^1^
*c* = 7.6269 (1) Å	*T* = 296 K
*V* = 3125.11 (5) Å^3^	Block, colourless
*Z* = 3	0.28 × 0.15 × 0.14 mm
*F*(000) = 2340	

### Data collection

**Table:**

Bruker SMART APEXII CCD area-detector diffractometer	1995 independent reflections
Radiation source: fine-focus sealed tube	1649 reflections with *I* > 2σ(*I*)
Graphite monochromator	*R*_int_ = 0.035
φ and ω scans	θ_max_ = 29.8°, θ_min_ = 1.9°
Absorption correction: multi-scan (*SADABS*; Bruker, 2008)	*h* = −30→30
*T*_min_ = 0.179, *T*_max_ = 0.338	*k* = −30→30
14898 measured reflections	*l* = −10→10

### Refinement

**Table:**

Refinement on *F*^2^	Primary atom site location: structure-invariant direct methods
Least-squares matrix: full	Secondary atom site location: difference Fourier map
*R*[*F*^2^ > 2σ(*F*^2^)] = 0.027	Hydrogen site location: inferred from neighbouring sites
*wR*(*F*^2^) = 0.061	H-atom parameters constrained
*S* = 1.06	*w* = 1/[σ^2^(*F*_o_^2^) + (0.0202*P*)^2^ + 17.0381*P*] where *P* = (*F*_o_^2^ + 2*F*_c_^2^)/3
1995 reflections	(Δ/σ)_max_ < 0.001
65 parameters	Δρ_max_ = 1.32 e Å^−^^3^
0 restraints	Δρ_min_ = −1.64 e Å^−^^3^

### Special details

**Table:**

Geometry. All e.s.d.'s (except the e.s.d. in the dihedral angle between two l.s. planes) are estimated using the full covariance matrix. The cell e.s.d.'s are taken into account individually in the estimation of e.s.d.'s in distances, angles and torsion angles; correlations between e.s.d.'s in cell parameters are only used when they are defined by crystal symmetry. An approximate (isotropic) treatment of cell e.s.d.'s is used for estimating e.s.d.'s involving l.s. planes.
Refinement. Refinement of *F*^2^ against ALL reflections. The weighted *R*-factor *wR* and goodness of fit *S* are based on *F*^2^, conventional *R*-factors *R* are based on *F*, with *F* set to zero for negative *F*^2^. The threshold expression of *F*^2^ > σ(*F*^2^) is used only for calculating *R*-factors(gt) *etc*. and is not relevant to the choice of reflections for refinement. *R*-factors based on *F*^2^ are statistically about twice as large as those based on *F*, and *R*- factors based on ALL data will be even larger.

### Fractional atomic coordinates and isotropic or equivalent isotropic displacement parameters (Å^2^)

**Table:**

	*x*	*y*	*z*	*U*_iso_*/*U*_eq_	
I1	0.082885 (13)	0.829523 (13)	0.17796 (4)	0.04164 (9)	
Cu1	0.04125 (3)	0.91805 (3)	0.12398 (9)	0.05416 (16)	
N1	−0.10312 (17)	0.76485 (17)	0.3103 (5)	0.0478 (8)	
H1N1	−0.1264	0.7232	0.3455	0.057*	
H2N1	−0.0616	0.7805	0.2909	0.057*	
N2	−0.19615 (15)	0.78569 (16)	0.2972 (4)	0.0394 (7)	
H1N2	−0.2067	0.8151	0.2721	0.047*	
C1	−0.12810 (18)	0.80754 (17)	0.2777 (5)	0.0330 (7)	
C2	−0.2500 (2)	0.7132 (2)	0.3356 (6)	0.0489 (10)	
H2A	−0.2945	0.7111	0.3573	0.073*	
H2B	−0.2363	0.6970	0.4375	0.073*	
H2C	−0.2549	0.6834	0.2375	0.073*	
S1	−0.07031 (4)	0.89448 (4)	0.21477 (14)	0.0388 (2)	

### Atomic displacement parameters (Å^2^)

**Table:**

	*U*^11^	*U*^22^	*U*^33^	*U*^12^	*U*^13^	*U*^23^
I1	0.04018 (14)	0.03935 (13)	0.05508 (16)	0.02713 (11)	0.00274 (11)	0.00505 (11)
Cu1	0.0380 (3)	0.0348 (2)	0.0945 (4)	0.0218 (2)	−0.0005 (3)	0.0007 (3)
N1	0.0340 (16)	0.0398 (17)	0.071 (2)	0.0197 (14)	0.0127 (16)	0.0209 (17)
N2	0.0316 (14)	0.0353 (15)	0.0534 (19)	0.0182 (13)	0.0053 (13)	0.0115 (14)
C1	0.0318 (16)	0.0306 (16)	0.0369 (17)	0.0157 (13)	0.0033 (14)	0.0031 (13)
C2	0.0305 (17)	0.042 (2)	0.063 (3)	0.0101 (16)	0.0039 (18)	0.0181 (19)
S1	0.0266 (4)	0.0264 (4)	0.0635 (6)	0.0133 (3)	0.0024 (4)	0.0039 (4)

### Geometric parameters (Å, º)

**Table:**

I1—Cu1	2.5379 (5)	N2—C1	1.317 (4)
Cu1—S1^i^	2.3164 (10)	N2—C2	1.449 (5)
Cu1—S1	2.3210 (10)	N2—H1N2	0.8028
Cu1—S1^ii^	2.6057 (13)	C1—S1	1.735 (3)
Cu1—Cu1^iii^	3.0264 (9)	C2—H2A	0.9600
Cu1—Cu1^ii^	3.0264 (9)	C2—H2B	0.9600
N1—C1	1.313 (4)	C2—H2C	0.9600
N1—H1N1	0.8316	S1—Cu1^iv^	2.3164 (10)
N1—H2N1	0.8039	S1—Cu1^iii^	2.6057 (13)
			
S1^i^—Cu1—S1	98.22 (5)	C1—N2—C2	124.5 (3)
S1^i^—Cu1—I1	122.56 (3)	C1—N2—H1N2	113.8
S1—Cu1—I1	120.95 (3)	C2—N2—H1N2	121.2
S1^i^—Cu1—S1^ii^	102.80 (4)	N1—C1—N2	120.7 (3)
S1—Cu1—S1^ii^	102.67 (4)	N1—C1—S1	119.5 (3)
I1—Cu1—S1^ii^	106.80 (3)	N2—C1—S1	119.7 (3)
S1^i^—Cu1—Cu1^iii^	118.09 (3)	N2—C2—H2A	109.5
S1—Cu1—Cu1^iii^	56.49 (3)	N2—C2—H2B	109.5
I1—Cu1—Cu1^iii^	118.39 (2)	H2A—C2—H2B	109.5
S1^ii^—Cu1—Cu1^iii^	47.86 (3)	N2—C2—H2C	109.5
S1^i^—Cu1—Cu1^ii^	56.52 (3)	H2A—C2—H2C	109.5
S1—Cu1—Cu1^ii^	118.03 (3)	H2B—C2—H2C	109.5
I1—Cu1—Cu1^ii^	119.84 (2)	C1—S1—Cu1^iv^	115.65 (12)
S1^ii^—Cu1—Cu1^ii^	47.96 (3)	C1—S1—Cu1	115.53 (12)
Cu1^iii^—Cu1—Cu1^ii^	85.08 (3)	Cu1^iv^—S1—Cu1	123.88 (5)
C1—N1—H1N1	126.2	C1—S1—Cu1^iii^	98.60 (12)
C1—N1—H2N1	116.1	Cu1^iv^—S1—Cu1^iii^	75.63 (3)
H1N1—N1—H2N1	117.6	Cu1—S1—Cu1^iii^	75.55 (3)
			
C2—N2—C1—N1	−7.1 (6)	Cu1^iii^—Cu1—S1—C1	92.76 (14)
C2—N2—C1—S1	174.9 (3)	Cu1^ii^—Cu1—S1—C1	154.78 (14)
N1—C1—S1—Cu1^iv^	171.7 (3)	S1^i^—Cu1—S1—Cu1^iv^	57.15 (8)
N2—C1—S1—Cu1^iv^	−10.3 (4)	I1—Cu1—S1—Cu1^iv^	−166.73 (4)
N1—C1—S1—Cu1	15.6 (4)	S1^ii^—Cu1—S1—Cu1^iv^	−48.03 (7)
N2—C1—S1—Cu1	−166.4 (3)	Cu1^iii^—Cu1—S1—Cu1^iv^	−61.20 (5)
N1—C1—S1—Cu1^iii^	93.6 (3)	Cu1^ii^—Cu1—S1—Cu1^iv^	0.83 (8)
N2—C1—S1—Cu1^iii^	−88.4 (3)	S1^i^—Cu1—S1—Cu1^iii^	118.35 (4)
S1^i^—Cu1—S1—C1	−148.90 (13)	I1—Cu1—S1—Cu1^iii^	−105.53 (3)
I1—Cu1—S1—C1	−12.78 (15)	S1^ii^—Cu1—S1—Cu1^iii^	13.17 (4)
S1^ii^—Cu1—S1—C1	105.92 (14)	Cu1^ii^—Cu1—S1—Cu1^iii^	62.03 (4)

Symmetry codes: (i) −*y*+1, *x*−*y*+2, *z*; (ii) *x*−*y*+1, *x*+1, −*z*; (iii) *y*−1, −*x*+*y*, −*z*; (iv) −*x*+*y*−1, −*x*+1, *z*.

### Hydrogen-bond geometry (Å, º)

**Table:**

*D*—H···*A*	*D*—H	H···*A*	*D*···*A*	*D*—H···*A*
N1—H1*N*1···I1^v^	0.83	2.95	3.744 (3)	161
N1—H2*N*1···I1	0.80	2.90	3.698 (4)	173
N2—H1*N*2···I1^iv^	0.80	2.95	3.756 (3)	177

Symmetry codes: (iv) −*x*+*y*−1, −*x*+1, *z*; (v) −*y*+2/3, *x*−*y*+4/3, *z*+1/3.
